# Biomechanical Morphing for Personalized Fitting of Scoliotic Torso Skeleton Models

**DOI:** 10.3389/fbioe.2022.945461

**Published:** 2022-07-19

**Authors:** Christos Koutras, Hamed Shayestehpour, Jesús Pérez, Christian Wong, John Rasmussen, Maxime Tournier, Matthieu Nesme, Miguel A. Otaduy

**Affiliations:** ^1^ Department of Computer Science, Universidad Rey Juan Carlos, Madrid, Spain; ^2^ Department of Materials and Production, Aalborg University, Aalborg, Denmark; ^3^ Orthopedics Department, University Hospital of Hvidovre, Hvidovre, Denmark; ^4^ AnatoScope SA, Montbonnot-Saint-Martin, France

**Keywords:** geometry fitting, biomechanical models, modeling of the torso, low-dose x-rays, scoliosis

## Abstract

The use of patient-specific biomechanical models offers many opportunities in the treatment of adolescent idiopathic scoliosis, such as the design of personalized braces. The first step in the development of these patient-specific models is to fit the geometry of the torso skeleton to the patient’s anatomy. However, existing methods rely on high-quality imaging data. The exposure to radiation of these methods limits their applicability for regular monitoring of patients. We present a method to fit personalized models of the torso skeleton that takes as input biplanar low-dose radiographs. The method morphs a template to fit annotated points on visible portions of the spine, and it relies on a default biomechanical model of the torso for regularization and robust fitting of hardly visible parts of the torso skeleton, such as the rib cage. The proposed method provides an accurate and robust solution to obtain personalized models of the torso skeleton, which can be adopted as part of regular management of scoliosis patients. We have evaluated the method on ten young patients who participated in our study. We have analyzed and compared clinical metrics on the spine and the full torso skeleton, and we have found that the accuracy of the method is at least comparable to other methods that require more demanding imaging methods, while it offers superior robustness to artifacts such as interpenetration of ribs. Normal-dose X-rays were available for one of the patients, and for the other nine we acquired low-dose X-rays, allowing us to validate that the accuracy of the method persisted under less invasive imaging modalities.

## 1 Introduction

Adolescent idiopathic scoliosis (AIS) is a complex spinal deformity that can lead to serious deterioration of quality of life and functional impairment. Its prevalence has been often documented as up to 5% (Konieczny et al., 2013; Yılmaz et al., 2020; Thomas et al., 2021), but recent work points to a prevalence of even 12% (Sung et al., 2021).

Severe cases are treated through complex surgery, and using braces is the method of choice to halt or delay the progression of the deformity with the ultimate goal of avoiding surgery (Kaelin, 2020). Scoliosis braces are used widely and effectively, but their design process could be optimized further. Most of the design choices are made through prototyping and physical experiments; computational design methods have been researched (Cobetto et al., 2016; Vergari et al., 2016), but they have not been adopted in practice.

The main challenge for the adoption of computational methods for the design of scoliosis braces is the personalization of biomechanical models of the torso (Kuroki, 2018). This personalization involves two aspects: mechanical characterization and geometric fitting; the latter is the focus of this work. A patient-specific model of the torso requires fitting the position and orientation of the bones in the torso. Correct fitting of the spine is crucial for correct evaluation of scoliosis. But a patient-specific model of the torso skeleton for brace design also requires correct fitting of the rib cage and the sternum, as they play an important role in the transformation of brace forces to the spine.

Before describing the novelty of our proposal, we discuss three lines of related work. First, we cover the general theme of geometric fitting, with the main methodologies. Second, we focus on our target problem, geometric fitting of the torso skeleton. And third, we pay attention to the mechanical modeling of the full torso, as it is a key ingredient of our proposalas it is a key part of our approach.

### 1.1 Background/Geometric Fitting

Geometric fitting refers to the problem of fitting a well-defined surface representation to a set of sparse measurements. This problem has been thoroughly studied, as it is an integral part of important applications, such as creating 3D models from scanner data. The various approaches to geometric fitting differ in the first place in terms of their input data. Common choices include dense point clouds coming from scanners (Li et al., 2008), volume images such as CT-scans (Gill et al., 2012), or sparse landmarks (Wang et al., 2021).

The solution methods for geometric fitting are also very diverse. For rigid objects, the most common approach is rigid template registration, which finds the rigid transformation of a template that minimizes a fitting cost (Zitová and Flusser, 2003). The same approach can be extended to non-rigid objects, balancing the fitting cost with a template deformation cost (Li et al., 2008). This is the approach we follow, but we propose novel fitting and template deformation costs applied to the torso. When the target object belongs to a family of objects and many examples are available, the template can also be parameterized using statistical shape modeling (Heimann and Meinzer, 2009). For medical volume data, geometric fitting is also connected to image segmentation (Haralick and Shapiro, 1985), which employs methods from all the families mentioned above.

### 1.2 Background/Geometric Fitting of the Torso Skeleton

The design of personalized torso skeleton models (either of a full torso skeleton or just some part) contributes to multiple clinical analyses and design applications. In addition to scoliosis brace design, which is the target application of this work, other applications include improving the assessment of the progression of diseases such as AIS, or increasing the success rate of spinal needle injection. Due to all these applications, a large effort has been devoted to the design of geometric fitting methods for the design of personalized torso skeleton models.

The first main approach involves the segmentation and registration of bones on volumetric image data. Rak et al. (2019) used neural networks and star convex cuts to segment the complete spine on MRI data. Kim et al. (2021) performed automatic detection and segmentation of lumbar vertebrae from X-ray images, and Di Angelo et al. (2021) proposed an automatic method for feature segmentation of thoracic and lumbar vertebrae. Gill et al. (2012) performed lumbar spine registration using CT and ultrasound data sets. Yip et al. (2014) developed an articulated registration algorithm for the full skeleton registration using CT scans, and Forsberg et al. (2013) performed a model-based registration for the assessment of spinal deformities in idiopathic scoliosis using CT scans as well. While CT scans provide rich information of the spine and the full torso, they also suffer limitations for scoliosis treatment. First, they are acquired in the prone or supine position, which is not well suited for scoliosis brace design, as they underestimate the scoliotic curve and deform the body envelope. Second, for adolescent patients with a developing spine, frequent acquisition of CT scans would require excessive radiation exposureresult in high accumulated radiation exposure.

The second main approach involves fitting a template model to input data. When data is sparse and/or noisy, the use of a template increases the robustness of the resulting model. Campbell and Petrella (2015) developed an automated method for landmark identification and finite element (FE) modeling of the lumbar spine using a template model. Humbert et al. (2009) proposed a 3D reconstruction of the spine from biplanar X-rays using parametric models based on transversal and longitudinal inferences. D evedzic et al. (2012) developed a 3D parametric model and simulator of the human spine for biomedical engineering education and scoliosis screening, and Gajny et al. (2019) developed a quasi-automatic 3D reconstruction of the full spine from low-dose biplanar X-rays based on statistical inferences. However, neither of these methods include the rib cage.

Many studies tried to reconstruct the rib cage in addition to the spine, following a template-based approach. Nankali et al. (2017) used CT scans and Cheriet et al. (2007) used two postero-anterior radiographs (the standard at 0° and another one at 20°) and one lateral radiograph. Other methods have focused their effort on reducing the radiation on the patient, and reconstructing the rib cage from biplanar radiographs (Mitton et al., 2008; Jolivet, Sandoz, Laporte, Mitton, Skalli; Seoud et al., 2009; Dworzak et al., 2010; Grenier et al., 2013; Aubert et al., 2016). Seoud et al. (2009), Mitton et al. (2008) and Grenier et al. (2013), after reconstructing the spine, estimated the mid lines of the ribs and reconstructed the full rib cage. These methods are slow for clinical use, as they need 30–40 min of manual effort per patient. Dworzak et al. (2010) used statistical shape models to facilitate the fully automated detection of objects in images. However, their initial solution must be similar to the actual solution in order to ensure robustness, especially when using *in vivo* data sets with poor quality. Jolivet et al. (Jolivet, Sandoz, Laporte, Mitton, Skalli) proposed an alternative method based on a geometric rib cage representation, but it needs the annotation of multiple landmarks, even in areas which are not clearly visible. Aubert et al. (2016) proposed a 3D reconstruction of the rib cage geometry from biplanar radiographs using a statistical parametric model. Then they improved their method, making landmark selection more user friendly and robust (Vergari et al., 2018). Shayestehpour et al. (2021) presented an articulated spine and rib cage kinematic model, which is able to attain scoliotic postures.

These studies provide interesting insights, but their processes could be optimized even further. Previous works morph only a part of the torso skeleton, such as the spine (Campbell and Petrella, 2015; Humbert et al., 2009; D evedzic et al., 2012; Gajny et al., 2019), or are too slow to be used in clinical settings (Mitton et al., 2008; Seoud et al., 2009; Grenier et al., 2013). Other works use input data which require considerable radiation, such as CT scans or multiple radiographs (Cheriet et al., 2007; Nankali et al., 2017). Additionally, the rest of the rib cage reconstruction methods use EOS radiographs (Aubert et al., 2016; Vergari et al., 2018; Jolivet, Sandoz, Laporte, Mitton, Skalli), which are not available in most clinical settings. An interesting alternative would be to use low-dose biplanar X-rays, which emit around one-eighth of the radiation of standard X-rays (Wong et al., 2021). However, previous methods (Aubert et al., 2016; Vergari et al., 2018; Jolivet, Sandoz, Laporte, Mitton, Skalli) would not be applicable to low-dose X-rays, as they need the annotation of various points in areas which are not clearly visible in low-dose X-rays, such as the rib cage.

### 1.3 Background/Mechanical Modeling of the Torso

Biomechanical modeling of the spine has received much attention, with mainly two different approaches. One approach follows the Finite Element Method (FEM) ((Wang et al., 2014) provides a survey). Several FEM models have been developed for the cervical (Lasswell et al., 2017), lumbar (Xu et al., 2017; Dong et al., 2020), or the thoracic spine (Aroeira et al., 2018). While these methods are potentially accurate, they require careful estimation of model parameters for personalized design applications. Nevertheless, many FEM models have been used for the design of personalized AIS braces (Gignac et al., 2000; Nie et al., 2009). Several works have also evaluated the effectiveness of these FEM methods (Cobetto et al., 2017; Vergari et al., 2020; Guy et al., 2021).

The second approach for biomechanical modeling of the spine uses a simpler but more efficient multibody system. de Zee et al. (2007) developed a generic rigid-body model of the lumbar spine. Ignasiak et al. (2015) extended this work and presented a multibody thoracolumbar spine model with articulated rib cage. Schmid et al. (2020) developed musculoskeletal full-body models including the thoracolumbar spine for children. Bayoglu et al. (2019) developed a multibody muscoloskeletal model of the human spine in order to study spinal loads. Le Navéaux et al. (2016) developed biomechanical models to analyze the effects of implant density and distribution on curve correction and the resulting forces on the vertebrae. Gould et al. (2021) published a literature review of computational modeling of the spine, focusing on both modeling approaches and healthy and scoliotic patients.

Hybrid models have combined both approaches, trying to combine their advantages. Dicko et al. (2015) developed a hybrid lumbar spine model containing rigid bodies, FEM and contact mechanics. Koutras et al. (2021) developed a comprehensive model of the torso, including the spine, the rib cage, and soft tissue.

Biomechanical models of the torso could be used as template models for geometric fitting. However, the models cannot be accurate before the geometry is personalized, hence it remains to understand what elements they should include.

### 1.4 Overview and Contributions

**FIGURE 1:**
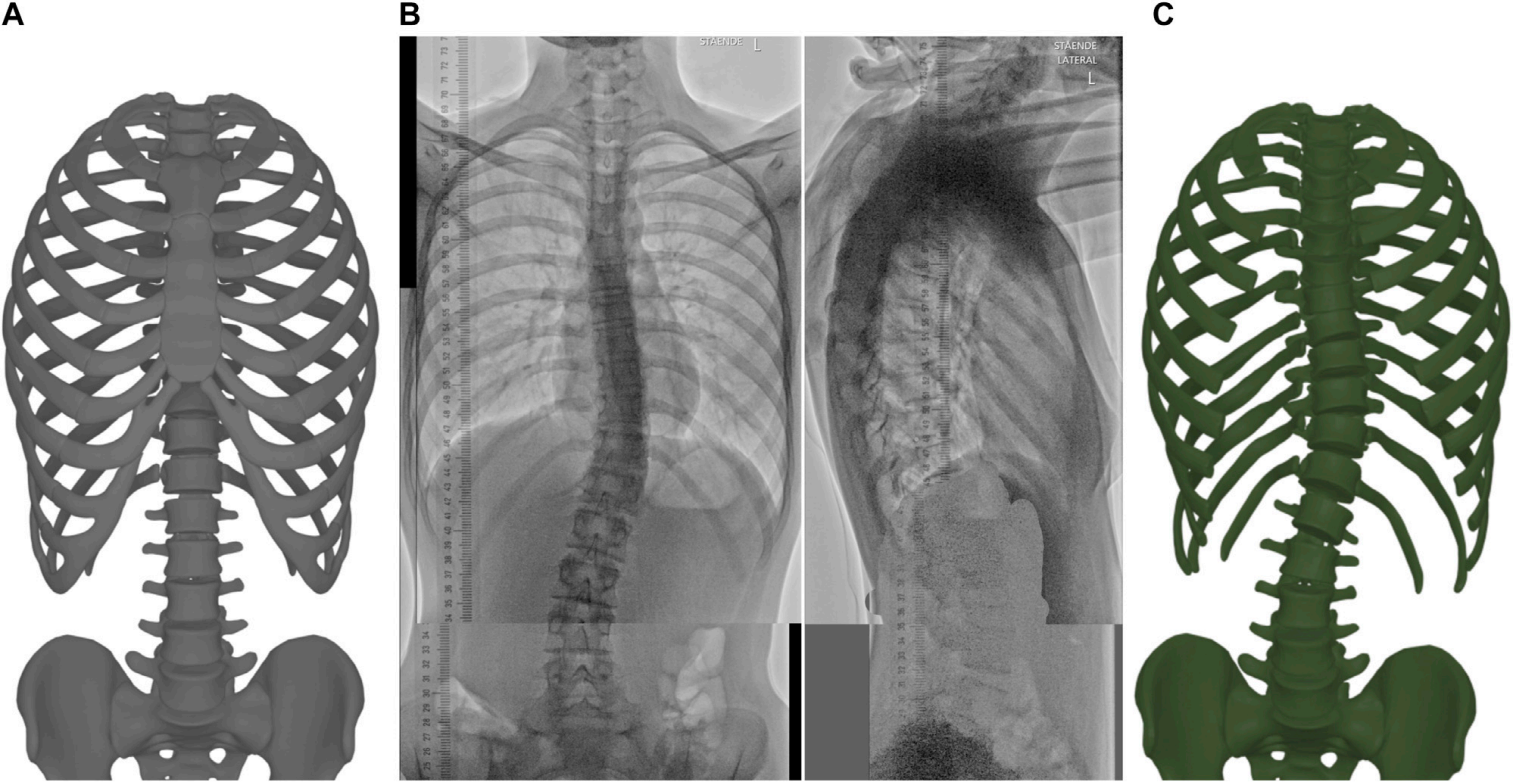
The proposed method takes as input a template model of the torso skeleton **(A)** and biplanar low-dose radiographs **(B)**, and outputs the morphed torso skeleton **(C)**. Here we hide the sternum and the costal cartilages to clearly show the spine.

In this work, we propose a method for geometric fitting of personalized torso skeleton models for AIS patients, as outlined in Figure 1. The resulting model includes the skeletal structure of the torso that plays a role in the transmission of forces from scoliosis braces to the spine, i.e., the spine itself, the pelvis, the rig cage, and the sternum. Therefore, the model becomes a key ingredient for patient-specific computer-aided design of braces. The proposed method uses as input biplanar low-dose X-ray radiographs, hence it can be easily adopted in the regular checkup procedure of AIS patients.

We propose to fit the torso skeleton geometry following a template morphing approach, which combines a biomechanical model with fitting targets. The biomechanical model of the full torso acts as a regularizer, allowing robust reconstruction of areas with noisy information, such as the ribs. Most importantly, we find that including the soft tissue in the biomechanical model is fundamental for this robustness. We show how the inclusion of soft tissue improves quantitative fitting metrics, but also qualitative robustness, e.g., preventing self-collisions in the model.

Section 2 describes the input data required by the method. In addition to the template model and the biplanar radiographs, the method requires manual annotation to calibrate the global scale of the radiographs and to determine the configuration of the spine. This annotation process is simple and fast.

Section 3 describes the morphing algorithm. First, it initializes the global scale of the torso geometry. Second, and most importantly, it morphs the template by solving an optimization problem. This optimization balances two goals. One goal consists of fitting the configuration of vertebrae that are clearly identified in the input radiographs. The other goal is a regularizer that minimizes the deformation of the template. We propose to use a biomechanical model of the torso as regularization, which naturally penalizes deformations that require higher biomechanical forces. As a result, the morphing algorithm produces configurations that require little biomechanical effort for bones that are hardly visible in the radiographs, such as the ribs. We have found that it is important to account for the effect of soft tissue in the biomechanical model. Adding a soft tissue model improves the fitting quality, but most importantly it improves the robustness of morphing, preventing issues such as collisions of bones.

Section 4 describes the experiments and validation. We have tested the proposed morphing algorithm on an initial cohort of ten (potential) AIS patients; further testing would be necessary to confirm clinical applicability. Normal-dose X-rays were available for one of the patients, and we captured low-dose X-rays for the other nine patients. We have measured several anatomical parameters on the resulting torso skeleton models, showing that accuracy is high and consistent across normal-dose and low-dose data. We have also confirmed that the regularization using the soft-tissue model avoids robustness issues. Finally, we have compared our method to previous work, showing superior performance. While the accuracy of our method is comparable to previous work on normal-dose X-rays, our method is more robust and it is applicable to low-dose X-rays with the same accuracy, while previous work is not.

## 2 Methods/Input Data

This section discusses the necessary inputs to the morphing method. These include the template torso geometry, the patient radiographs, and the manual annotation of the radiographs for the definition of the target spine configuration. The section also describes the calibration of the scale of the radiographs.

### 2.1 Template Geometry

The personalized torso model consists of the skeletal system that plays a role in scoliosis and/or participates in the transmission of forces from scoliosis braces to the spine. In this way, the personalized torso skeleton model can be used for computer design of personalized scoliosis braces (Gignac et al., 2000; Nie et al., 2009). We design this torso skeleton model by morphing a template model to match the specific anatomy of each patient.

The template model consists of the bones of the thoracic and lumbar spine, the rib cage, the sternum, the pelvis, the costal cartilages and the soft tissue. More details about the template model are explained in Section 3.3. The template torso skeleton geometry corresponds to a healthy (non-scoliotic) adolescent female.

### 2.2 Radiographs

To capture a patient’s torso skeleton, we use biplanar radiographs, as they are readily available as part of the regular check-up of scoliotic patients, and they provide sufficient visibility of bone structures. We use low-dose radiographs, which minimize the radiation applied to patients. Specifically, we used a DelftDI D2RS system with fluoroscopic exposure (Wong et al., 2021).

When capturing the radiographs, the patients are asked to put their hands over their head, to obtain better visibility of the torso. The resulting images show part of the pelvis, the lumbar and the thoracic spine, part of the cervical spine and the rib cage, i.e., the portion of the skeleton that we wish to fit. The vertical range of the low-dose X-ray machine is limited; therefore, taller patients required two radiographs per plane, four in total. The radiographs were stitched together by clinical experts. In the rest of the manuscript, the stitched radiographs are considered as a single radiograph.

### 2.3 Scale Calibration

The calibration of radiographs implies understanding the parameters of the perspective projection of the subject’s anatomy onto the radiograph image. For calibrated imaging systems, such as EOS, this information is easy to obtain. In our setting, however, we use an uncalibrated imaging system. Fortunately, as the depth range of the anatomy is narrow with respect to the distance to the projection plane, we can leverage the assumption of a weak-perspective projection (Kadoury et al., 2007). Then the imaging process can be approximated as a scaled orthographic projection on each frontal and sagittal view, respectively (Xu and Zhang, 1996; Yoo et al., 2004). The weak perspective projection is considered an appropriate assumption when the depth range is less than 10% of the average depth (Thompson and Mundy, 1987). In our setting, the worst case occurs in the frontal image, with an average depth (distance from the center of projection to the mid-line of the spine) of approximately 115 cm, and a depth range (half of the spine’s lateral width) of approximately 8.5 cm. This amounts to a maximum error bound of 7.8% on the estimation of the positions of vertebrae.

Following the assumption of weak perspective projection, calibration amounts to finding the scale of each radiograph. We do this by installing a ruler on the projection plane, next to the patient’s spine. This ruler is visible in both frontal and sagittal radiographs, and it allows us to easily measure the size of the torso in all three dimensions. We use this information to define the global scale of the torso model as we discuss later in Section 3.1. Due to the perspective projection, the ruler does not appear continuous in the stitched radiographs, but this does not affect the estimated scale.

### 2.4 Annotation of Transformations

The two radiographs provide two planar views of the torso skeleton. However, most of the skeleton is hardly visible. Landmarks or features of the desired anatomical elements cannot be identified on both radiographs; therefore, we cannot rely on triangulation to extract 3D information of specific points. Instead, we opt to extract from the radiographs the following information: 2D positions and in-plane rotations of the vertebrae and pelvis, and from those their 3D position and orientation; and the Cobb angle of the spine, which is correlated to its axial rotation (Takahashi et al., 2007; Easwar et al., 2011; Mao et al., 2012). The rest of the geometry of the torso skeleton is reconstructed using our morphing algorithm, as described in Section 3.

For each vertebra, we annotate its center on both radiographs, and we use this information to define a target position $x_{i}^{*}$ in 3D. We use the longitudinal and lateral position from the frontal radiograph, and the sagittal position from the sagittal radiograph. Thus, we convert two pairs of 2D coordinates into the 3D coordinates of the vertebra centers.

For each vertebra, we also annotate the lateral and sagittal rotation. We do this by identifying the orientation of the line passing through the upper end-plate of the vertebra, by marking two points on this end-plate on the frontal and sagittal radiographs, respectively. For visualization, in the images in the paper we show only one point, on a line parallel to the end-plate and passing through the center of the vertebra. For the pelvis, we also define a lateral rotation, based on the line that passes through the top points of the left and right iliac crests.

We define a target orientation $R_{i}^{*}$ per vertebra, by combining rotations on three orthogonal planes: the lateral and sagittal rotations, based on the annotations described above, and the axial rotation. The axial rotation of vertebrae cannot be inferred from the low-dose radiographs, due to their limited visual quality. However, several studies have documented a correlation between the axial rotation of the apex vertebra (i.e., the vertebra furthest from the line passing from the head to the pelvis on the frontal plane) and the Cobb angle, in particular an apical axial rotation of 52% of the Cobb angle (Takahashi et al., 2007; Easwar et al., 2011; Mao et al., 2012). We measure the Cobb angle on the frontal radiograph, we compute accordingly the axial rotation of the apex vertebra, and then we linearly interpolate this rotation along the spine between the apex vertebra and the first top and bottom neutral vertebrae (i.e., those with no visible axial rotation on the radiographs).

The target orientations and positions of the vertebrae and the pelvis, $\left\{R_{i}^{*},x_{i}^{*}\right\}$ constitute the input information to the morphing algorithm.

## 3 Methods/Morphing Algorithm

This section describes the morphing of the template torso skeleton to match a patient’s specific anatomy, based on the input data described in Section 2. The morphing proceeds in two steps. The first step applies a global scale to the template torso, to match the overall dimensions of the patient’s torso. The second step optimizes the position and orientation of the different bones. We formulate an optimization that balances two competing goals. On one hand, a cost function *E*_*fitting*_ measures the fitting of the bones that are annotated in the radiographs. On the other hand, a cost function *E*_*deformation*_ measures the deformation away from the template torso. By balancing these two goals, we obtain a resulting torso skeleton that matches accurately the patient’s anatomy and is smooth and free of artifacts. The section proceeds by describing the scale step, the fitting function, the deformation function, and finally the solution to the resulting optimization problem.

### 3.1 Global Scale

We start the morphing process by applying a global scale to the template torso, such that it fits the overall size of the patient’s torso. We apply a non-uniform scale along the longitudinal, sagittal, and transversal axes. For the longitudinal scale, we use the length of the spine between T1 and L5 vertebrae. For the transversal scale, we use the maximum width between the sixth pair of ribs. Unfortunately, the image quality on the sagittal X-ray is not sufficient to identify an independent sagittal scale, hence we initialize the template model with the sagittal scale equal to the longitudinal scale.

### 3.2 Fitting Function

As described in Section 2.4, we characterize the configuration of the patient’s torso using the transformations of vertebrae, obtained from the radiographs. Then, we define a fitting cost function *E*_*fitting*_ that measures the error between the vertebrae in the output torso and the vertebrae of the patient. Specifically, for each vertebra, we define the target rotation and position, $R_{i}^{*}$ and $x_{i}^{*}$ , and the (initially unknown) output rotation and position, **R**_*i*_ and **x**_*i*_. We quantify the relative rotation using an axis angle representation $\Delta \theta_{i}=AxisAngle\left(R_{i}^{T}\;R_{i}^{*}\right)$ , and the relative translation simply as the difference vector $\Delta x_{i}=R_{i}^{T}\;\left(x_{i}-x_{i}^{*}\right)$ . Both the relative rotation and translation are expressed in the local frame of the vertebra, which is convenient for the definition of anisotropic weights. The fitting cost function is obtained by summing squared error terms across all vertebrae:

$$E_{\mathrm{fitting}}=\underset{i}{\sum }\Delta \theta_{i}^{T}\;\text{diag}\left(k_{\theta ,i}\right)\;\Delta \theta_{i}+\Delta x_{i}^{T}\;\text{diag}\left(k_{x,i}\right)\;\Delta x_{i}.$$ (1)

The terms $k_{x,i}=\left(k_{x,i},k_{y,i},k_{z,i}\right)$ and $k_{\theta ,i}=\left(k_{\alpha ,i},k_{\beta ,i},k_{\gamma ,i}\right)$ represent vectors of error weights. The axes are locally aligned for each vertebra as: *x* translation along the frontal axis, *y* translation along the longitudinal axis, *z* translation along the sagittal axis, *α* flexion/extension, *β* axial rotation, *γ* lateral bending. We weigh the various error terms differently, both across vertebrae and across axes of the same vertebra, depending on the confidence of the measurements of the patient’s spine. After some experiments, we settled the weights listed in Table 1. The weights of flexion/extension and axial rotation are lower for the first 6 thoracic vertebra, as the low-dose radiographs are less clear in that part of the spine. On the contrary, the apical vertebra provides valuable information; therefore, we want to match its position and orientation accurately, and we select a much higher weight.

**TABLE 1:** Error weights (in kN/m for translation, N/rad for rotation) for the fitting function (Eq. 1).

Vertebrae	${\bar{k}}_{x}$	${\bar{k}}_{y}$	${\bar{k}}_{z}$	${\bar{k}}_{\alpha }$	${\bar{k}}_{\beta }$	${\bar{k}}_{\gamma }$
Apical	100	100	100	1e12	1e12	1e12
Apical to Neutral	100	100	100	1e5	1e5	1e5
T1 to T6	100	100	100	100	100	1e3
Others	100	100	100	100	100	1e5

We also include Table 2 with joint stiffness values of a biomechanical model of an average torso (which we actually use for the deformation function described next in Section 3.3). As evidenced in the tables, we used uniform translation weights across all the vertebrae, a value similar or lower than the translation stiffness of the biomechanical model. With this choice, the inter-vertebral connections of the template model are preserved, while the vertebrae are pulled to their target positions. We used non-uniform rotation weights, very high for the apical and neighboring vertebrae (which are clearly visible in the radiographs), and lower for the rest. We also used relatively higher lateral bending stiffness, as lateral rotation is clearly visible on the frontal radiograph, except for vertebrae T1 to T6, which are less clear.

**TABLE 2:** Joint stiffness values (in kN/m for translation, N/rad for rotation) for the biomechanical model of the deformation function.

Vertebrae	${\bar{k}}_{x}$	${\bar{k}}_{y}$	${\bar{k}}_{z}$	${\bar{k}}_{\alpha }$	${\bar{k}}_{\beta }$	${\bar{k}}_{\gamma }$
Thoracic segment	262	1720	262	154	137	154
Lumbar segment	245	1720	245	143	498	149

### 3.3 Deformation Function

The radiographs provide only partial information about the patient’s torso skeleton, as the image of some bones is noisy or even not visible. To fill in this missing information and ensure a robust output torso geometry, we rely on the template model as regularizer. We define a cost function *E*_*deformation*_ that measures the deviation between the output torso and the template torso, and we add it to the fitting cost (Eq. 1).

The deviation between the output and template torsos can be interpreted as a deformation of the torso; therefore, we propose to quantify this deformation using a biomechanical model of the torso. Intuitively, this regularization approach favors torso deformations that require small forces in reality, e.g., flexion of the spine vs. separation of vertebrae.

The use of a biomechanical model of the torso as regularizer suggests the question of what degree of biomechanical accuracy is required. We choose a multibody model of the torso skeleton, with bones modeled as rigid bodies, joints modeled as compliant 6D joints, and a homogeneous finite-element model of soft tissue coupled to the bones (Koutras et al., 2021). We model bones as rigid bodies because this assumption is on par with the degrees of freedom we wish to estimate, i.e., the bone transformations of a template model. We model joints as compliant as this choice allows some vertebra separation for accurate matching of reliable vertebrae according to the fitting function (Allard et al., 2007). We use default stiffness parameters corresponding to average subject values (Choi and Zheng, 2005; Ignasiak et al., 2015), as it is not possible to obtain patient-specific information, and default average values suffice to provide the desirable regularization effect. Finally, we choose to model the soft tissue surrounding the bones, as it produces a stronger regularization on the rib cage. We have compared the deformation model with and without soft tissue, and the addition of soft tissue increases the overall fitting quality and the robustness of the model, as we discuss in detail later in Section 4.4. Notably, the addition of soft tissue prevents interpenetrations at the ribs, which occur when soft tissue is not included in the model.

By adding soft tissue to the bone skeleton structure of the torso, the degrees of freedom (DoFs) **x** of the morphing problem consist now of the bone transformations and the displacements of soft-tissue nodes. The deformation cost function is nothing else but the elastic energy of the biomechanical model, which depends on the complete set of DoFs, *E*_*deformation*_(**x**). We refer to (Koutras et al., 2021) for full details on the parameterization and computation of the elastic energy of the biomechanical model.

### 3.4 Solution to the Optimization

Given the fitting and deformation cost functions, we wish to find the DoFs **x** that minimize the summed cost. Formally, this is formulated as:

$$x=\mathrm{arg min}E_{\mathrm{fitting}}\left(x\right)+E_{\mathrm{deformation}}\left(x\right).$$ (2)

Note that the fitting cost function of each vertebra can be interpreted as the elastic energy of a 6D spring connecting the output vertebra to the patient’s vertebra. Therefore, the full optimization (Eq. 2) can be regarded as an energy minimization problem. The energy minimum corresponds to a static equilibrium condition, given by the internal forces of the torso $f_{\mathrm{deformation}}=\frac{\partial E_{\mathrm{deformation}}}{\partial x}$ and the external forces produced by the 6D springs of the fitting function, $f_{\mathrm{fitting}}=\frac{\partial E_{\mathrm{fitting}}}{\partial x}$ :

$$f_{\mathrm{fitting}}\left(x\right)+f_{\mathrm{deformation}}\left(x\right)=0.$$ (3)

As the optimization requires solving a mechanical equilibrium problem, it is possible to use off-the-shelf simulation engines for this purpose. In particular, we have used the SOFA simulation framework (Allard et al., 2007). SOFA solves the equilibrium problem using dynamic relaxation with kinetic damping (Volino, Magnenat-Thalmann). To initialize the optimization, we translate the template such that L5 is located at its target position.

## 4 Results

The proposed torso morphing method was applied to a cohort of ten female potential AIS patients, ranging from 10 to 17 years old, with a mean Cobb angle of 17° and a standard deviation of 10. The subjects were selected because they had to be screened for scoliosis based on previous diagnosis or examination. Subject #2 was not considered an AIS patient after all. In order to proceed with the study, we obtained oral and written consent from the patients according to national Danish guidelines and the Helsinki Declaration, and with approval of the local ethics committee at University Hospital of Hvidovre (No. H-17034237).

**FIGURE 2:**
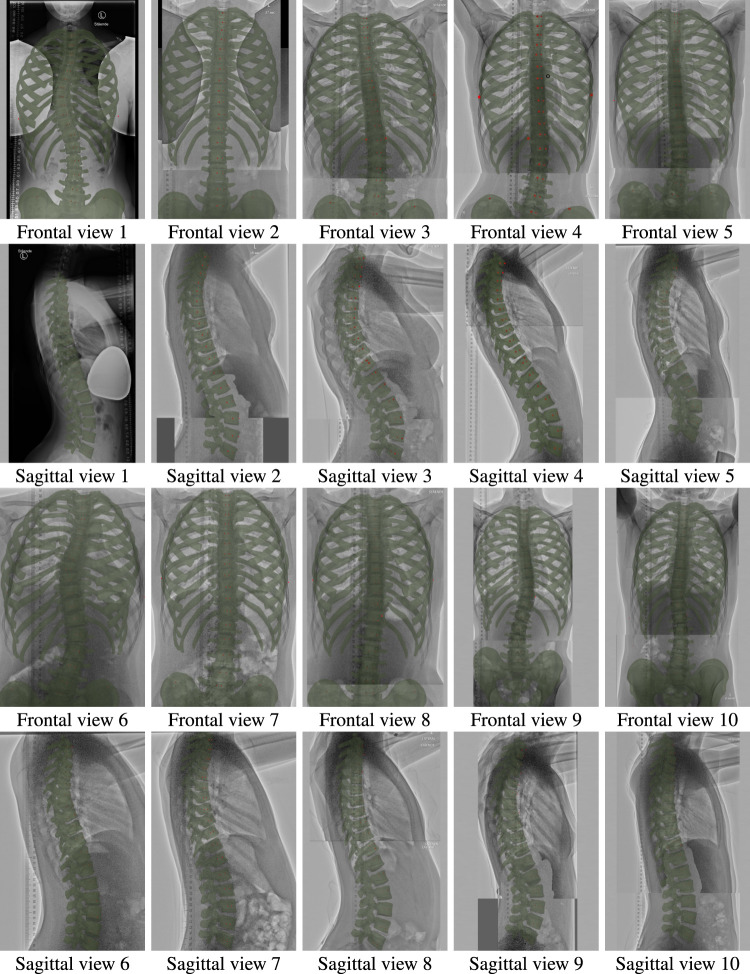
Frontal and sagittal views of all ten patients of the study, with the morphed models overlaid on the input radiographs.

For one particular validation case, we used normal-dose radiographs of a patient from a previous study (Shayestehpour et al., 2021), obtained with a digital Carestream DRX-Evolution system with automatic exposure control (see Patient 1 in Figure 2). For all other nine patients, we used the low-dose X-ray system described in Section 2.2.

To validate the proposed biomechanical morphing method, we evaluate a set of clinical metrics on the resulting torso skeleton models for all patients, and we compare them with direct measurements from the radiographs. The results provide evidence of the accuracy of our method, but further validation on a larger cohort of patients is necessary before application in clinical settings. We also validate that the accuracy of the method is comparable using low-dose vs. normal-dose radiographs. Finally, we compare our method to previous work, and we confirm that it achieves superior performance. While the accuracy of our method is comparable to previous work on normal-dose X-rays, our method is more robust and it is applicable to low-dose X-rays as well.

The section starts with a description of the metrics used for validation. Then, it discusses the qualitative evaluation of the morphing method for all patients, the comparison between normal-dose and low-dose X-rays, and the comparison to previous work. The section concludes with a discussion of timings for the complete modeling process, including manual annotation and the optimization-based morphing algorithm.

### 4.1 Evaluation Metrics

To evaluate the quality of the resulting torso skeleton models, we have measured the following clinically relevant metrics. We chose to use these metrics as they provide information about both frontal and sagittal planes. Scoliosis is a 3D spinal deformity; therefore, correction in the sagittal plain is equally important as in the frontal one (Salmingo et al., 2014). For a more detailed description and definition of the metrics, the reader may refer to (Shayestehpour et al., 2021).

• Main thoracic Cobb angle (MT Cobb) is the standard measurement used to quantify spine deformities in the case of scoliosis. It is defined in the thoracic part of the spine.
• Thoracolumbar/lumbar Cobb angle (TL/L Cobb) is the Cobb angle in the lumbar or thoracolumbar part of the spine.
• Translation of thoracic apex (AVTThorax) is the linear distance in the transversal axis between the pelvis and the apex of the thoracic spine.
• Translation of lumbar apex (AVTLumbar) is the linear distance in the transversal axis between the pelvis and the apex of the lumbar spine.
• Lumbar lordosis angle (LL) is the angle defined between the superior endplate of L5 and the inferior endplate of L1 in the sagittal plane.
• Thoracic kyphosis angle (TK) is the angle defined between the superior endplate of T1 and the inferior endplate of T12 on the sagittal plane.
• Pelvis lateral rotation (PLR) is the rotation of the pelvis in the frontal plane.
• Apical vertebral body-rib ratio (AVBR) is the ratio of the linear measurements between the borders of the apical vertebra and the right and left chest walls.
• Rib vertebra angle (RVA) is the angle between a line parallel to the endplate of a vertebra and a line passing from the mid-neck to the mid-head of the corresponding rib. It is defined on every vertebra level *i*, and on both the left and right sides of the patient. We denote specific vertebra values as RVA_*Li*_ and RVA_*Ri*_, and average values as RVA_*i*−*j*_.
• Rib vertebra angle difference (RVAD) is the difference between the right and left RVA, and it is defined on every vertebra level *i*. We denote specific vertebra values as RVAD_*i*_, and average values as RVAD_*i*−*j*_.
• Rib hump (RH) is the linear distance between the left and right posterior rib prominences along the antero-posterior axis, on each vertebra level *i*. We denote specific vertebra values as RH_*i*_, and average values as RH_*i*−*j*_.
• Rib spread difference (RSD_*i*−*j*_) is the difference between the left and right intercostal distances at vertebra levels *i* and *j*. It is measured along the longitudinal axis.
• Sternal notch translation along the longitudinal axis (STNT_*y*_), measured relative to T10.
• Sternal notch translation along the transversal axis (STNT_*x*_), measured relative to T10.

### 4.2 Error Evaluation

Figure 2 illustrates the morphed torso skeletons for all participants of the study. The figure shows frontal and sagittal views of the results, drawn semitransparent in green, on top of the original radiographs.

The metrics defined in the previous section can be manually measured on frontal and sagittal views of the torso. Then, to evaluate the error of our morphing algorithm, we generate 2D frontal and sagittal views of the resulting 3D torso models, and we compare the metrics to those measured directly on the input radiographs.

Table 3 lists the errors in the metrics, averaged across all nine patients with low-dose radiographs. As special cases, thoracic and lumbar Cobb angle were not measured on all patients, only on those for which these metrics were relevant (8 patients in the case of thoracic Cobb angle, and 5 patients in the case of lumbar Cobb angle). Most of the errors are within measurement tolerances (e.g., 5° for Cobb angle (Langensiepen et al., 2013)). The table does not show the rib hump RH, the thoracic kyphosis angle TK, or the sternal notch translations STNT, as they could not be measured on low-dose radiographs.

**TABLE 3:** Error on the clinical evaluation metrics for the torso models resulting from our morphing.

X-rays	RVA_*R*6_	RVA_*L*6_	RVA_*R*7_	RVA_*L*7_	RVA_*R*8_	RVA_*L*8_	RVA_*R*9_	RVA_*L*9_	RVA_*R10*_
Low-dose Mean	3 *deg*	2.2 *deg*	2.8 *deg*	2.6 *deg*	6.4 *deg*	3.5 *deg*	7.3 *deg*	5.2 *deg*	8.8 *deg*
Low-dose STD	2.8 *deg*	2.2 *deg*	3.7 *deg*	2.0 *deg*	5.0 *deg*	1.8 *deg*	1.8 *deg*	3.8 *deg*	3.2 *deg*
Normal	6 *deg*	4 *deg*	3 *deg*	5 *deg*	4 *deg*	0 *deg*	2 *deg*	13 *deg*	5 *deg*
	**RVA_*L10*_**	**RVA_6−10_**	**RVAD_6_**	**RVAD_7_**	**RVAD_8_**	**RVAD_9_**	**RVAD_10_**	**RVAD_6−10_**	
Low-dose Mean	5.3 *deg*	4.7 *deg*	3.6 *deg*	2.8 *deg*	4.8 *deg*	4.5 *deg*	3.6 *deg*	3.9 *deg*	
Low-dose STD	4.0 *deg*	1.3 *deg*	1.9 *deg*	5.1 *deg*	4.0 *deg*	4.2 *deg*	3.4 *deg*	3.2 *deg*	
Normal	5 *deg*	4.7 *deg*	10 *deg*	11 *deg*	4 *deg*	2 *deg*	10 *deg*	7.4 *deg*	
	**RSD_7−12_**	**AVBR**	**MT Cobb**	**TL/L Cobb**	**AVTThorax**	**AVTLumbar**	**LL**	**PLR**	
Low-dose Mean	1.8 *mm*	0.05	1.7 *deg*	1.2 *deg*	1.3 *mm*	1.8 *mm*	3 *deg*	0.05 *deg*	
Low-dose STD	1.97 *mm*	0.03	2.05 *deg*	0.57 *deg*	1.03 *mm*	0.87 *mm*	3.18 *deg*	0.16 *deg*	
Normal	1 *mm*	0.23	2 *deg*	0 *deg*	1 *mm*	3 *mm*	2 *deg*	0 *deg*	

We show separately the average errors and standard deviations of the low-dose cases, and the errors of the normal-dose case.

### 4.3 Normal-Dose vs. Low-Dose X-Rays

One of the goals of our method is to overcome the low visual quality of low-dose radiographs, thanks to the regularization provided by the biomechanical model. To test this goal, we compared the fitting quality with normal-dose radiographs of one patient from a previous study (Shayestehpour et al., 2021), and low-dose radiographs for all nine new patients.

**FIGURE 3:**
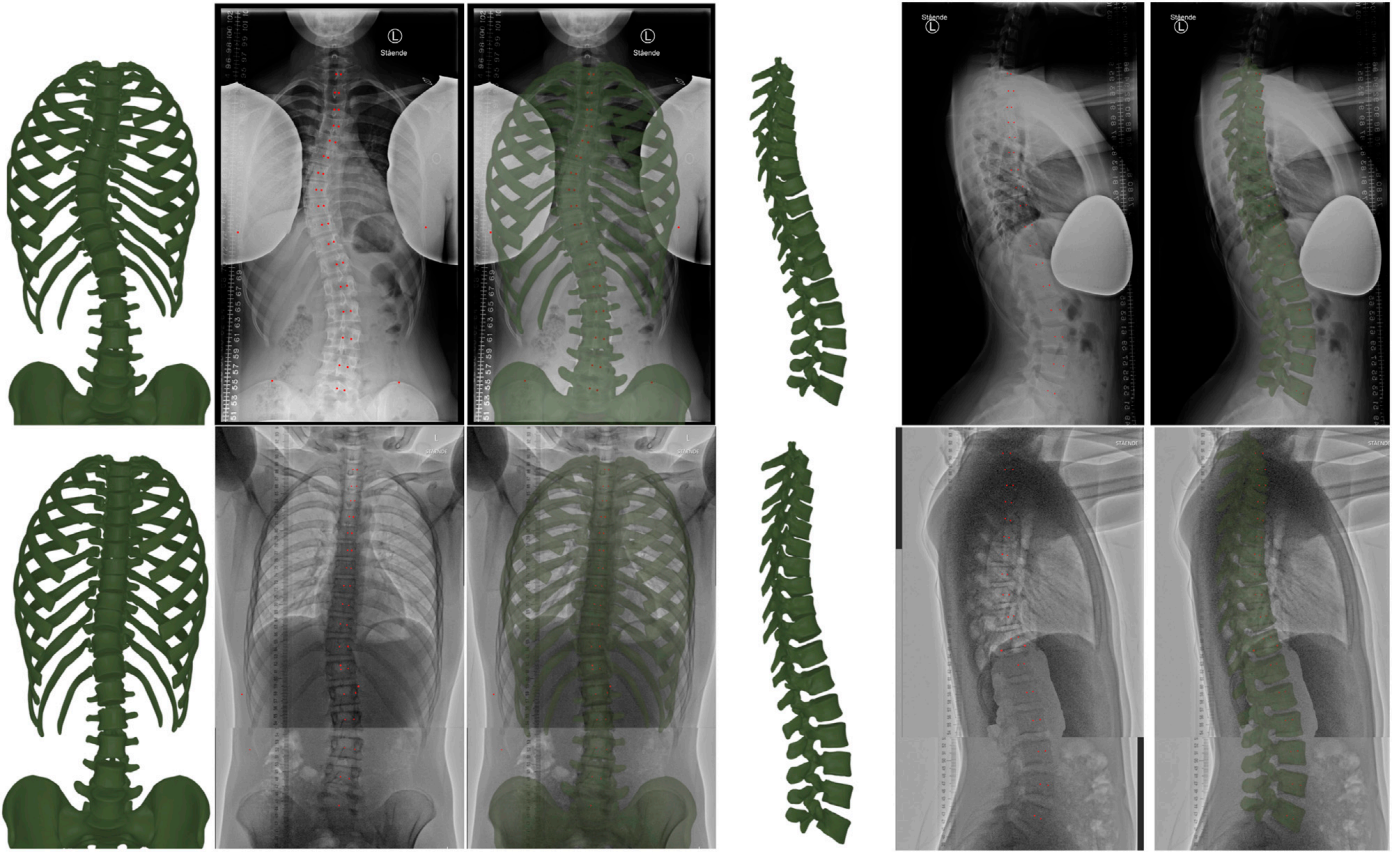
From left to right, the figure illustrates the torso skeleton model resulting from our method, the input radiograph, and an overlay (with the resulting model drawn green semi-transparent), in both the frontal and sagittal planes. The top row corresponds to a case with normal-dose radiographs, and the bottom row to a case with low-dose radiographs. Note that we hide the sternum and cartilages in the frontal images, as well as the ribs in the sagittal images, to provide a clear view of the spine. The red dots are the points selected during manual annotation.

Figure 3 compares qualitatively the resulting model for normal-dose and low-dose radiographs. The figure shows the input radiographs, the resulting model, and an overlay, on both the frontal and sagittal planes. We hide the sternum and the cartilages on the frontal view, as well as the rib cage on the sagittal view, to provide a clear image of the spine.

Table 3 compares quantitatively the errors on the fitting metrics listed earlier, for normal-dose and low-dose radiographs. As evidenced by the data, the errors for the low-dose cases are often lower than for the normal-dose case. However, note that the average Cobb angle was 17° for the low-dose cases, and 33° for the normal-dose case; therefore, the improved quality on the low-dose cases is due to reduced scoliosis on average, and the net fitting quality is similar on normal-dose and low-dose cases.

### 4.4 Comparison to Previous Work

We have compared our results to the morphing method of (Shayestehpour et al., 2021) on the normal-dose case, as it is shared by both studies. As discussed in Section 1.2, the method of (Shayestehpour et al., 2021) also uses a biomechanical model as regularizer, but based on the musculoskeletal structure of the torso, without the soft tissue. In addition, it uses several clinical skeletal metrics (15 in total, including the last 9 in Table 4, from MT Cobb to STNT_*x*_) as fitting objectives.

**TABLE 4:** Comparison of fitting results between our method and (Shayestehpour et al., 2021), on the normal-dose case (patient 1). The columns indicate, from left to right, clinical metrics measured on radiographs, metrics obtained using (Shayestehpour et al., 2021), metrics with our method, and error improvement thanks to our method (positive values indicate that our method outperforms (Shayestehpour et al., 2021)).

	X-ray	Shayestehpour et al. (2021)	Ours	Improvement		X-ray	Shayestehpour et al. (2021)	Ours	Improvement
RH_5_	14 *mm*	12 *mm*	20 *mm*	−4 *mm*	RVAD_7_	42 *deg*	51 *deg*	40 *deg*	+7 *deg*
RH_8_	33 *mm*	30 *mm*	37 *mm*	−1 *mm*	RVAD_8_*	35 *deg*	51 *deg*	39 *deg*	+12 *deg*
RH_10_	39 *mm*	25 *mm*	22 *mm*	−3 *mm*	RVAD_9_	26 *deg*	16 *deg*	15 *deg*	−1 *deg*
RH_5,8,10_**	—	—	—	−2.7 *mm*	RVAD_10_	11 *deg*	18 *deg*	1 *deg*	−3 *deg*
RVA_*R*6_	46 *deg*	48 *deg*	52 *deg*	−4 *deg*	RVAD_6−10_**	—	—	—	+2.8 *deg*
RVA_*L*6_	101 *deg*	94 *deg*	97 *deg*	+3 *deg*	RSD_7−12_	20 *mm*	14 *mm*	21 *mm*	+5 *mm*
RVA_*R*7_	50 *deg*	47 *deg*	47 *deg*	0 *deg*	AVBR	0.64	0.86	0.87	−0.01
RVA_*L*7_	92 *deg*	98 *deg*	87 *deg*	+1 *deg*	MT Cobb	33 *deg*	33 *deg*	35 *deg*	−2 *deg*
RVA_*R*8_*	46 *deg*	42 *deg*	42 *deg*	0 *deg*	TL/L Cobb	24 *deg*	24 *deg*	24 *deg*	0 *deg*
RVA_*L*8_*	81 *deg*	93 *deg*	81 *deg*	+12 *deg*	AVTThorax	24 *mm*	24 *mm*	25 *mm*	−1 *mm*
RVA_*R*9_	47 *deg*	55 *deg*	45 *deg*	+6 *deg*	AVTLumbar	7 *mm*	7 *mm*	10 *mm*	−3 *mm*
RVA_*L*9_	73 *deg*	71 *deg*	60 *deg*	−11 *deg*	LL	44 *deg*	44 *deg*	46 *deg*	−2 *deg*
RVA_*R10*_	41 *deg*	48 *deg*	46 *deg*	+3 *deg*	TK	30 *deg*	30 *deg*	36 *deg*	−6 *deg*
RVA_*L10*_	52 *deg*	67 *deg*	47 *deg*	+10 *deg*	PLR	0 *deg*	0 *deg*	0 *deg*	0 *deg*
RVA_6−10_**	—	—	—	+2 *deg*	STNT_*y*_	165 *mm*	165 *mm*	164 *mm*	−1 *mm*
RVAD_6_	55 *deg*	46 *deg*	45 *deg*	−1 *deg*	STNT_*x*_	15 *mm*	15 *mm*	18 *mm*	−3 *mm*

* In the apical vertebra. ** Average improvement.

As illustrated in Table 4, with our method we obtain comparable accuracy. However, our method has two significant advantages. One is that it works robustly with low-dose radiographs. The method of (Shayestehpour et al., 2021), on the other hand, uses as input clinical metrics (such as the thoracic kyphosis angle TK or the sternal notch translations STNT_*y*_ and STNT_*x*_) that must be manually annotated on the input radiographs. These metrics can be measured with reasonable accuracy with normal-dose X-rays, but they are highly error-prone with low-dose X-rays, as the upper thoracic vertebrae are poorly visible on the sagittal radiographs, and the sternum is practically invisible on most frontal radiographs.

**FIGURE 4:**
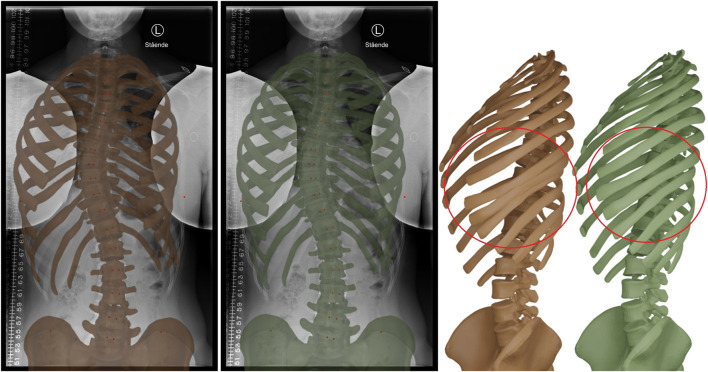
Morphing result without (brown) and with (green) soft tissue in the biomechanical model. The inclusion of the soft tissue provides a stronger regularization effect on the rib cage, and prevents interpenetrations at the ribs, as highlighted on the right.

The other significant advantage is offered by the inclusion of soft tissue in the biomechanical model. Figure 4 compares a morphing result of our method with and without soft tissue. Note how the lack of soft tissue leads to interpenetrations at the ribs, which are eliminated when soft tissue is added. The method of (Shayestehpour et al., 2021) suffers similar interpenetrations, as evidenced in their results. By adding the soft tissue, we also increased significantly the fitting quality. The errors of the rib hump RH, rib vertebra angles RVA, and rib vertebra angle differences RVAD on the normal-dose case were reduced from 13 mm, 10.8° and 20° to 9 mm, 4.7 and 7.4°, respectively, when soft tissue was included.

### 4.5 Timings

The proposed method simplifies the task of manual annotation with respect to previous work, as it only requires stitching the pairs of radiographs and annotating the width of the rib cage and positions and orientations of vertebrae. We have timed the task of manual annotation for the input cases, and this time was on average 2 min 40 s for the frontal radiograph and 5 min for the sagittal radiograph, including the stitching.

The optimization-based morphing is faster than the annotation task. In our examples, it always took less than 1 minute. Thus the total torso reconstruction time is below 6 min per patient if stitching of radiographs is not needed, and below 8 min per patient when stitching is needed.

## 5 Discussion

Low-dose X-rays offer beneficial properties for regular checkup of AIS patients, as they reduce the exposure to radiation. However, they come with challenges for the creation of computer models of the torso and hence the adoption of computational solutions for, e.g., brace design. The spine is visible with sufficient detail in low-dose radiographs, but other parts of the torso skeleton, such as the rib cage, which are necessary for computational brace design, are hardly visible.

Our results demonstrate that a template-based morphing solution provides a robust way of reconstructing hardly visible areas of the torso geometry, and thus enables the creation of personalized torso models even using low-dose radiographs as input. The key to the proposed template-based morphing approach is to use a biomechanical model of the torso for regularization of the torso’s deformation. In the computation of the target torso skeleton geometry, we balance a fitting function that tries to match the spine with a deformation function that minimizes the deformation away from the template. Notably, regularization does not require a personalized biomechanical model. A model with default rest state and default parameters suffices to provide the required regularization effect, while retaining enough flexibility to match the fitting data. Furthermore, the morphing algorithm works by solving a regular static equilibrium problem, and it does not require a specialized solver.

In our experiments, we have investigated key elements of the biomechanics model. In particular, we find that including the soft tissue in the model ensures a robust regularizer that avoids artifacts such as interpenetrations of the ribs. Our model also considers soft constraints at all joints, which allow some translation, e.g., between vertebrae, to accommodate possible scale errors in the fitting data.

Despite its benefits, the proposed approach suffers some limitations. One is that it reconstructs the transformations of bones, but not their shapes. Another one is that the fitting quality degrades at areas with little data such as the lower ribs. Both limitations share common causes, as the proposed method only alters the transformations of template bones, but it is unable to change the shape of individual bones. This limitation is not present in EOS systems, but our method is available for a broader range of hospitals without access to EOS systems.

A possible direction of future work is to modify the template and use a parametric statistical model instead. As the method is used to create personalized models, the model results could be leveraged to define a parametric statistical model. Then, the parametric model could substitute the fixed template in the fitting step, and thus improve its versatility and accuracy.

The participants in the study were mostly patients with mild or moderate scoliotic curve. As a result, there is not sufficient evidence of how the proposed pipeline behaves on severe scoliotic cases. Broader analysis of severe cases is postponed to future work, as well as evaluation on a larger cohort of patients to validate clinical applicability. Note that we do not target patients who require spine surgery, only braces, and then Cobb angle may remain below 40–45° (Maruyama and Takeshita, 2009; Zhu et al., 2017). The largest Cobb angle among the patients in our cohort was 33°, and it would be necessary to cover multiple cases in the 30–45° range. As mentioned earlier, perhaps a statistical model could also extend the applicability of the method, but it is unclear how to build a statistical model for severe cases.

Finally, an additional limitation of our work is that it requires manual input for landmark annotation, and this could lead to errors. While the use of soft constraints improves the tolerance to annotation errors, it is worth investigating automated methods that would substitute manual annotation.

For applications such as scoliosis brace design, personalization of torso models should be covered from two angles: geometric fitting, as done in this work, but also mechanical response. We plan to extend our work by personalizing the mechanical response of torso skeleton models. This requires designing an experimental procedure for the acquisition of training data, and designing robust and accurate parameter estimation methods.

## Data Availability

The original contributions presented in the study are included in the article/supplementary material, further inquiries can be directed to the corresponding author.
